# Effect of Oxycodone hydrochloride combined with Dexmedetomidine on quality of recovery and stress response after general anesthesia in patients who had Laparoscopic Cholecystectomy

**DOI:** 10.12669/pjms.37.5.3959

**Published:** 2021

**Authors:** Guo-rui Wang, Qian Wu, Wen-ping Liu, Yu-mo Jing

**Affiliations:** 1 Guo-rui Wang, Department of Anesthesiology, Cangzhou Hospital of Integrated TCM-WM • Hebei, Cangzhou, Hebei, 061000, P.R. China; 2 Qian Wu, Department of Anesthesiology, Cangzhou Hospital of Integrated TCM-WM • Hebei, Cangzhou, Hebei, 061000, P.R. China; 3 Wen-ping Liu, Department of Anesthesiology, Cangzhou Hospital of Integrated TCM-WM • Hebei, Cangzhou, Hebei, 061000, P.R. China; 4 Yu-mo Jing, Department of Anesthesiology, Cangzhou Hospital of Integrated TCM-WM • Hebei, Cangzhou, Hebei, 061000, P.R. China

**Keywords:** Dexmedetomidine, Laparoscopic Cholecystectomy, Oxycodone hydrochloride

## Abstract

**Objective:**

To explore the effect of oxycodone hydrochloride combined with dexmedetomidine on the recovery quality and stress response during anesthesia in patients undergoing laparoscopic cholecystectomy (LC).

**Methods:**

Ninety patients who had LC in Cangzhou Hospital of Integrated TCM-WM of Hebei from December 2016 to December 2019 were selected and divided into dexmedetomidine group (DEX group), oxycodone hydrochloride group (Q group), dexmedetomidine + oxycodone hydrochloride group (DQ group) by a random number table method, with 30 cases in each group. At the time before anesthesia induction (T0), and immediately (T1), 1 min (T2), 10 min (T3) and 30 minutes (T4) after extubation, the general vital signs of three groups were observed, and plasma cortisol (COR), epinephrine (E), norepinephrine (NE) and blood glucose (GLU) were measured. The spontaneous respiration recovery time, wake-up time, VAS score of each time period after extubation, extubation quality score, and adverse event rate were recorded.

**Results:**

The vital signs at each time point of extubation, recovery time of spontaneous respiration, wake-up time, and extubation quality of DQ group were better than those of DEX group and Q group (P<0.05). The incidence of agitation, VAS score at T2 and T3, plasma concentrations of Cor, E, NE and Glu at T1, T3 and T4 in DQ group were significantly lower than those in Q group and DEX group (P<0.05).

**Conclusion:**

Oxycodone hydrochloride combined with dexmedetomidine can improve the recovery quality and reduce stress response in patients with LC after anesthesia, and can be safely used in patients with LC.

## INTRODUCTION

Laparoscopic surgery is a common surgical technique that is characterized by clear operative field of vision, less tissue trauma, etc.^1^ Compared with the traditional open cholecystectomy, laparoscopic cholecystectomy (LC) has the advantages of shorter operation time, less amount of bleeding, less trauma and faster recovery. However, the pneumoperitoneum established during the operation may affect the cardiopulmonary function of the patients to some extent, result in intraoperative fluctuations in vital signs, and also significant activation of stress response.^2^,^3^ The recovery period from general anesthesia is a high-risk stage of anesthesia, with high possibility of inducing restlessness and choking cough in the patient due to the decreased depth of anesthesia, endotracheal intubation, aspiration of sputum, wound pain.^4^ It may cause cardiovascular stress reactions such as increased blood pressure (BP), increased heart rate (HR), and increased oxygen levels in the myocardium.^5^,^6^ Oxycodone hydrochloride is a semisynthetic opioid receptor agonist with dual excitatory effect of μ and κ receptor, used in clinical settings that can reduce stress response during extubation, yet with unideal effect so far.^7^ Besides, dexmedetomidine is an α2-adrenergic receptor agonist^8^ with common application in clinical practice. Oxycodone hydrochloride and dexmedetomidine have been reported to possess complementary roles in pharmacology.^9^ The present study explored the effects of oxycodone hydrochloride combined with dexmedetomidine on the recovery quality and stress response of patients underwent LC during the recovery period of general anesthesia.

## METHODS

Ninety patients underwent elective LC under general anesthesia in Cangzhou Hospital of Integrated TCM-WM of Hebei from December 2016 to December 2019 were selected, randomly divided into dexmedetomidine group (DEX group), oxycodone hydrochloride group (Q group), dexmedetomidine + oxycodone hydrochloride group (DQ group), with 30 cases in each group. The sample size required for each group is calculated by the formula:


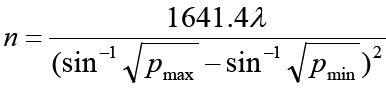


As shown in Table-I, there was no significant difference in gender, age, weight, operation time and other general information among the three groups (P>0.05). The study had been approved by the local Ethics Committee, and all patients signed the informed consent form.

**Table-I:** Comparison of general data of three groups of patients.

Groups	Cases (n)	Gender (male/female)	Age (years)	Body mass (Kg/m^2^)	ASA classification (I/II)	Operation time (min)	Intraoperative blood loss (ml)
DEX group	30	20/10	50.8±18.3	22.03±2.83	17/13	120.1±28.1	202.4±50.5
Q group	30	19/11	49.6±19.5	21.78±3.28	18/12	118.3±30.9	199.9±51.2
DQ group	30	21/9	50.6±18.9	21.85±3.16	18/12	122.2±29.3	205.9±45.9

### Ethical approval

The study was approved by the Institutional Ethics Committee of Cangzhou Hospital of Integrated TCM-WM of Hebei (No./066/01.0/2017), and written informed consent was obtained from all participants.

### Exclusion criteria:

1. Patients with significant dysfunction of important organs or chronic disease history.
2. Patients with acute and chronic infections such as hepatitis, tuberculosis and pneumonia.
3. Patients with nervous, mental and endocrine system diseases.
4. Patients with a history of opioid allergy.
5. Patients have a history of analgesics abuse or who have recently taken analgesics and sedatives.

Patients were informed to abstain from food and water before operation. Multifunctional monitor was applied to monitor arterial systolic pressure (SBP), diastolic blood pressure (DBP), mean arterial pressure (MAP), respiratory rate (RR), heart rate (HR), blood oxygen saturation (SPO_2_) and other indexes. The patient was intravenously induced anesthetized, and then connected to a ventilator after endotracheal intubation for mechanical ventilation. Dexmedetomidine was infused intravenously 15 minutes before anesthesia induction in DEX and DQ groups at an initial dosage of 0.8μg/kg, and then continuously pumped at the rate of 0.4μg.kg^-1^.h-1. After laparoscopy was removed, both group Q and group DQ were given oxycodone hydrochloride at 0.10 mg/kg via intravenous drip.

### Observational indexes:

1. Hemodynamic changes were recorded in the three groups of patients before anesthesia induction (T0), immediately after extubation (T1), one minute after extubation (T2), 10 minureafter extubation (T3), and 30 min after extubation (T4), respectively, including heart rate (HR), systolic blood pressure (SBP), diastolic blood pressure (DBP), blood oxygen saturation (SPO_2_).
2. The spontaneous respiration recovery time, wake-up time, extubation time, extubation quality score were recorded in the three groups.
3. The visual analogue scale (VAS) scores were recorded at T2, T3, one hour and two hour after extubation.
4. The incidence of restlessness, nausea and vomiting, respiratory depression, arrhythmia, drowsiness and other adverse events were observed in patients during the recovery period.

### VAS scoring standard

The pain was assessed and divided into 10 levels (0 point, no pain; 1~3 point (s), mild and tolerable pain; 4~6 points, moderate and tolerable pain that affected the sleep; and 7~10 points, the worst imaginable pain.

### Rating standard of restlessness

one point, restlessness and intense struggle; two points, frequent and active expression of discomfort; three points, quiet, with physical activity when stimulated; four points, slow response when called; five points, deep sleep and no response when called.

### Rating standard of extubation quality^10^

one point, no cough; two points, mild cough; three points, moderate cough; four points, severe cough for 5~10 times or breath holding; and five points, severe cough >10 times or laryngospasm.

An amount of 8ml of peripheral venous blood was collected at T0, T1, T3 and T4. The level of cortisol (Cor), epinephrine (E), norepinephrine (NE), and blood glucose (Glu) were detected.

### Statistical analysis

Statistical analysis of data was realized by using SPSS22.0 software. Measurement data was expressed by mean ± standard deviation (X±s) and analyzed by t test. Intra-group comparison used one-way analysis of variance of repeated measurement. The counting data was presented by X^2^ and analyzed using chi-square test. P<0.05 meant that the difference was statistically significant.

## RESULTS

There was no significant difference in HR, SBP, DBP and SPO_2_ of the three groups of patients at T0 (P>0.05). These indicators in the DQ group were more stable than those in the DEQ and Q groups (P<0.05). Detailed results are shown in Table-II. The recovery time of spontaneous breathing, recovery time, wake-up time, and extubation quality of patients in the DQ group were better than those in the DEX and Q groups, but without statistical difference (P>0.05), as shown in Table-III.

**Table-II:** Comparison of hemodynamics among the three groups of patients.

Groups	Cases	Indexes	T0	T1	T2	T3	T4
DEX group	30 cases	HP (times/min)	77.50±4.95	90.41±5.26*	89.40±5.21*	82.55±4.80*	83.19±4.13*
		SBP(mmHg)	116.38±10.25	141.38±13.45*	142.32±13.25*	123.38±11.20*	126.35±12.35*
		DBP(mmHg)	73.57±5.25	90.52±7.21*	90.56±7.14*	78.70±6.26*	79.53±6.77*
		SPO_2_ (%)_)_	99.59±0.18	90.38±0.23*	91.38±0.34*	98.50±0.20	99.55±0.15
Q group	30 cases	HP (times/min)	77.44±4.82	90.75±4.85*	90.40±5.21*	83.77±4.43*	84.22±5.61*
		SBP(mmHg)	115.28±10.85	142.52±15.81*	141.32±13.25*	123.93±13.67*	129.40±13.99*
		DBP(mmHg)	73.40±5.86	91.26±7.91*	91.56±7.14*	79.36±6.86*	80.53±6.73*
		SPO_2_ (%)_)_	99.59±0.18	90.38±0.23*	91.38±0.34*	98.50±0.20	99.55±0.15
DQ group	30 cases	HP (times/min)	77.41±4.76	83.22±4.58	83.40±4.21	77.72±4.60	79.61±4.81
		SBP(mmHg)	115.45±10.34	141.38±12.79	132.32±11.25	119.26±11.50	123.52±11.91
		DBP(mmHg)	73.36±5.84	79.59±6.56	80.95±6.34	75.27±6.02	76.12±6.25
		SPO_2_ (%)	99.59±0.17	98.38±0.23	98.38±0.34	98.50±0.20	99.55±0.15

***Note:*** Compared with DQ group,

* P<0.05.

**Table-III:** Comparison of spontaneous respiration recovery time, wake-up time, extubation time and extubation quality among the three groups of patients.

Groups	Cases (n)	Spontaneous respiration recovery time (min)	Wake-up time (min)	Extubation time (min)	Extubation quality (points)
DEX group	30	3.96±1.54	10.12 ±2.97	13.87±3.84	3.29±0.9
Q group	30	3.63±1.53	10.56 ±2.75	13.09±2.85	3.23±0.8
DQ group	30	3.52±1.65	11.43±3.72	13.20±3.96	3.21±0.5

At T2 and T3, the VAS score of DQ group was significantly lower than that of Q group and DEX group, with significant statistical difference (P<0.05; Table-IV). The incidence of restlessness in DQ group was lower than that in Q group and DEX group (P<0.05, Table-V). At T1, T3, and T4, the plasma concentrations of Cor, E, NE, and Glu in the DQ group were significantly lower than those in the Q and DEX groups (P<0.05, Table-VI).

**Table-IV:** Comparison of VAS scores among the three groups of patients at different time after extubation.

Groups	Cases (n)	T2	T3	1h after extubation	2h after extubation
DEX group	30	2.62±0.32*	2.91 ±0.51*	3.23±0.61	3.44±0.72
Q group	30	2.32±1.51*	2.85 ±0.62*	3.53±0.71	3.61±0.82
DQ group	30	1.83±0.31	2.21±0.40	3.00±0.50	3.15±0.48

***Note:*** Compared with DQ group,

* P<0.05.

**Table-V:** Comparison of the incidence of adverse events among the three groups of patients (n/%).

Groups	Cases (n)	restlessness	Nausea and vomiting	Respiratory depression	Arrhythmia	drowsiness
DEX group	30	8/26.67*	3/10	0	3/10	6/20
Q group	30	7/23.33*	2/6.67	0	2/6.67	5/16.67
DQ group	30	2/6.67	4/13.33	0	3/10	4/13.33

***Note:*** Compared with DQ group,

* P<0.05.

**Table-VI:** Comparison of the concentrations of Cor, E, NE and Glu among the three groups of patients (x±s).

Indexes	Groups	Cases	T0 (Before induction)	T1(At the end of extubation)	T3 (10min)	T4 (30min)
Cor (ng/ml)	DEX group	30	145.9±26.8	202.1±28.3*#	196.3±20.1*#	178.7±29.0*#
	Q group	30	141.0±21.9	200.6±24.2*#	197.4±29.7*#	179.35±22.3*#
	DQ group	30	140.2±31.7	154.7±29.0*	161.4±26.9*	160.9±29.1*
E (pg/ml)	DEX group	30	15.6±3.2	29.7±4.0*#	27.1±3.8*#	28.2±3.4*#
	Q group	30	15.0±3.9	28.9±3.1*#	28.1±4.8*#	27.2±4.0*#
	DQ group	30	15.2±3.5	21.0±3.5*	20.2±3.3*	20.3±3.0*
NE (pg/ml)	DEX group	30	340.1±102.1	491.7±105.6*#	448.5±99.1*#	450.3±97.5*#
	Q group	30	348.7±101.2	499.9±98.7*#	450.4±99.7*#	448.2±95.7*#
	DQ group	30	344.2±98.6	396.8±100.1*	381.9±92.7*	386.3±95.5*
Glu (mmol/l)	DEX group	30	5.4±0.5	12.6±0.8*#	11.9±0.8*#	11.9±0.6*#
	Q group	30	5.5±0.6	11.9±0.9*	11.9±0.7*#	11.7±0.5*#
	DQ group	30	5.5±0.5	9.8±0.7*	8.6±0.4*	8.5±0.4*

***Note:*** Compared with T0,

* P<0.05;

Compared with DQ group, #P<0.05.

## DISCUSSION

The recovery period from general anesthesia refers to a process of continuous degradation and elimination of the anesthetic drug, as well as reduction of anesthesia depth. Patients underwent LC may be stimulated by trauma, tracheal tube, urinary tube, drainage tube and other stimulants, and affected by residual anesthetic drugs during the recovery period from general anesthesia. It may lead to hemodynamic changes in these patients (e.g., increased BP, increased HR, decreased blood oxygen concentration and restlessness, etc.), and it can be primary explained by postoperative pain stimulation. Meanwhile, effective analgesia after extubation also plays an important role in reducing complications during post-anesthesia recovery.^11^,^12^

In clinical settings, drug intervention is a common approach to relieve the pain and stress response occurred during the recovery period. At present, there are many available drugs that can be selected in the recovery period of general anesthesia. However, there is uncertain analgesic and sedative effects of low-dose drugs, and high-dose drugs may induce higher risk of adverse reactions, which is not conducive to the recovery of anesthesia. It in turn significantly restricts the application of monotherapy in clinical practice. Oxycodone hydrochloride and dexmedetomidine are commonly used drugs during post-anesthesia recovery. However, it has not been reported with respect to the effect of their combined usage on the quality of extubation and stress response of patients underwent LC in the recovery period so far.

Oxycodone is a semi-synthetic opioid derived from the opium alkaloid thebaine that can work quickly, exhibiting good analgesic and sedative effects.^13^,^14^ However, it has also been reported that oxycodone at a higher dose can cause respiratory depression in patients.^15^,^16^ Furthermore, dexmedetomidine is a new type of α2-adrenergic receptor agonist with the effects of sedation, stress inhibition, and reduction of sympathetic activity.^18^ Nevertheless, it has a great negative impact on cardiopulmonary circulation, with a higher risk of inducing hypotension and bradycardia in the patient.^17^,^19^ Acting as analgesics and sedatives with different mechanisms, oxycodone hydrochloride and dexmedetomidine may act on different sites of pain reflex arc respectively, and thus exert a synergistic effect under a combined usage.^20^ In our study, it was observed that oxycodone or dexmedetomidine alone was not enough to reverse the hemodynamic changes caused by stress response in patients underwent LC during the recovery period from general anesthesia. Significantly, their combined use could exert a satisfactory analgesic effect but without influence in post-anesthesia recovery of patients in time.

Restlessness is manifested by the coexistence of excitement, restlessness and disorientation, which may cause severe consequences such as suffocation, bleeding at the surgical site, broken sutures on the incision, and urinary retention. It usually occurs about 15 minutes after extubation.^21^ How to avoid patient agitation during the recovery period is always the key to improving the quality of resuscitation. Oxycodone hydrochloride is such an agonist with dual excitatory effect of μ and κ receptor. It has a significantly lower incidence of nausea and vomiting than that of the specific μ receptor agonist, with the advantages of less restlessness, as well as no mental addiction, gastrointestinal inhibition and respiratory inhibition.^22^ Meanwhile, dexmedetomidine generates effects such as sedation, hypnosis, anti-anxiety, anti-stress and easy to wake up, which can relieve the bad mood and exert a good sedation effect in the patients.^23^ In terms of the lower incidence of restlessness in our study, it was speculated that oxycodone or dexmedetomidine alone was inadequate to inhibit restlessness during post-anesthesia recovery^24^, and the combination exhibited a better effect.

Stress response during the recovery period from general anesthesia involves changes in the nerve, endocrine, immunity and metabolism. The changes in the concentration of Cor, E, NE and Glu in plasma are recognized as sensitive indexes for evaluating the intensity of stress response^25^, and their secretion are consistent with the intensity of stress response.^26^ In this study, there was significant decrease in the concentrations of Cor, E, NE and Glu in the plasma of patients in DQ group at T2, T3 and T4. It supports that oxycodone hydrochloride combined with dexmedetomidine can reduce the systemic stress response and the secretion of vasoactive molecules in patients underwent LC during post-anesthesia recovery.

To sum up, oxycodone hydrochloride combined with dexmedetomidine can be effective to maintain a more stable hemodynamic status without effect on the extubation quality, which can be conducive to reducing pain response, restlessness and stress response in patients underwent LC.

### Limitations of the study

The combined application of these two drugs exerts a synergistic effect, while also reducing the side effects and adverse reactions caused by the two drugs alone, and can be safely applied to LC patients during the recovery period from general anesthesia. Of course, this study still has some limitations and shortcomings, such as insufficient sample size of each group, insufficient evaluation indicators, etc., which need to be further studied and improved in the future.

### Authors’ Contributions:

**GW andQW** designed this study and prepared this manuscript, and are responsible and accountable for the accuracy or integrity of the work.

**WL** collected and analyzed clinical data.

**YJ** significantly revised this manuscript.
